# Lessons Learned from the Design and Implementation of the Tuberculosis Free Nepal Initiative

**DOI:** 10.31729/jnma.8988

**Published:** 2025-05-31

**Authors:** Prajowl Shrestha, Gokul Mishra, Mukti Nath Khanal, Naveen Prakash Shah, Deepak Dahal, Barsha Thapa, Lok Raj Joshi, Namita Ghimire, Tom Wingfield

**Affiliations:** 1 National Tuberculosis Control Centre, Naya Thimi, Bhaktapur, Nepal; 2 Nayathimi, Bhaktapur; 3 World Health Organization, Pulchowk, Lalitpur, Nepal; 4 Save the Children International, Nepal; 5 Nepal Health Research Council, Ramshahpath, Kathmandu; 6 Departments of Clinical Sciences and International Public Health, Liverpool School of Tropical Medicine, Pembroke Place Liverpool L3 5QA UK; WHO Collaborating Centre in TB and Social Medicine, Karolinksa Institutet, Sweden, Norrbackagatan 4, 171 76; Tropical and Infectious Diseases Unit, Liverpool University Hospitals NHS Foundation Trust, Liverpool, L7 8YE, UK

**Keywords:** *directly observed treatment short-course*, *end tuberculosis*, *microplanning*, *tuberculosis free*, *tuberculosis*

## Abstract

**Introduction:**

Nepal has a persistently high burden of tuberculosis. Despite implementation of multiple interventions by the National tuberculosis Program, Nepal is not on track to achieve many of WHO's End tuberculosis Strategy targets.

**Methods:**

The National tuberculosis Control Centre developed a Google Sheet with key indicators to monitor the tuberculosis -Free Initiative across municipalities. Focal points recorded real-time data, ensuring transparency. National tuberculosis Control Centre compiled, analyzed, and interpreted the data to track progress, evaluate program outcomes, and support future planning.

**Results:**

The tuberculosis-Free Initiative achieved significant results in implementing municipalities. A total of 112 END TUBERCULOSIS Committees were formed at the municipal level, with over 1,000 ward-level committees engaged in tuberculosis microplanning. More than 56% of municipalities mobilized community-led monitoring groups, and 16 municipalities conducted annual social audits. tuberculosis-Free Volunteers facilitated screening in 53 municipalities. In 2023, innovative case-finding methods contributed significantly. The sputum courier system identified 1,790 Pulmonary bacteriologically confirmed tuberculosis cases, 554 cases were diagnosed via screening camps and door-to-door visits, and 222 cases through Primary healthcare centre Outreach Clinics. Additionally, 23 patient support groups, 32 youth groups, and 32 civil society organizations were mobilized, strengthening community participation. These efforts highlight the tuberculosis-Free Initiative's impact on enhancing case detection, community engagement, and tuberculosis control strategies.

**Conclusions:**

Developing local level ownership and accountability in the national tuberculosis response, ensuring high quality implementation through robust monitoring and evaluation, and generating and sustaining local resources, requires strong government leadership, advocacy, and capacity building. Within the implementing teams by the stakeholders, frequent initiative reviews, coaching, and mentoring support.

## INTRODUCTION

In 2021, an estimated 10.6 million people globally fell ill with tuberculosis (TB). Alarmingly, approximately 3 million cases were never notified, diagnosed, or treated, leading to 1.6 million deaths.^1^ One of the major challenges in reducing TB-related mortality and advancing efforts to end TB is the identification of missed cases, predominantly occurring in low- and middle-income countries (LMICs).

Nepal, a low-income country in the South East Asian region has demonstrated high TB treatment success rates (~90%). However, findings from the 2018/19 Nepal National TB Prevalence Survey showed significantly higher TB prevalence (416 per 100,000), incidence (245 per 100,000) and mortality (58 per 100,000) than previous estimated.^2^ These figures equates to approximately 69,000 incident TB cases annually, with half of these cases remaining undetected.^3^

**Figure 1:**
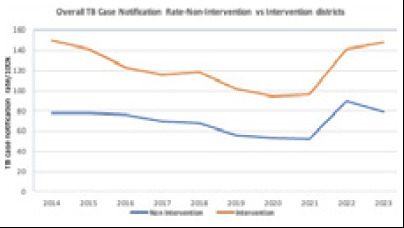
Overall TB Case Notification Rate (Non intervention vs intervention districts)

Nepal's National TB Program (NTP), supported by Global Fund, has introduced innovative interventions targeting 42 densely-populated districts covering 80% of the population. These interventions aim to identify missed TB cases, increase case notification rates, and enhance linkage to care. The remaining 35 less-populated districts receive standard care from domestic funding. Recent evaluations have illustrated Nepal's struggle to meet national and World Health Organization (WHO) End TB targets for TB incidence and mortality.^3^ Between 2014 and 2021, the TB case notification rate decreased by 25%. In 2022 and 2023, the rate increased, possibly related to the effects of the COVID pandemic and expansion of molecular diagnostic tool throughout the country and contributions of Sub-recipients (Figure 1).

Despite the TB Free Nepal Initiative and investment, trends in TB case notification rates were similar in intervention and non-intervention districts except in 2023.

In 2019, an external review emphasized sustaining Nepal's TB response through active engagement across all government levels. Challenges included tailoring interventions to district contexts, enhancing local government autonomy, capacity building, and involving TB-affected individuals, local stakeholders, and communities, especially to increase TB knowledge and address TB-stigma. COVID further compounded these challenges.

## METHODS

Interrupting TB transmission is crucial to end TB.^4^ The Zero TB initiative is a multisectoral collaborative dedicated to addressing TB disparities in megacities with high TB burden. Its successes have included fostering local authority involvement and equitable partnerships to enhance TB strategies.^5-6^

In 2021, the Government of Nepal launched the "TB Free Nepal Initiative" to end TB in Nepal by 2050. Aligned with current National Strategy Plan for TB (2021/22-2025/26),this initiative aims to empower local decentralised governments to manage their TB programs using local resources and strategies.^7^

Responding to the Nepal TB Prevalence Survey findings, the Initiative recognised the need for geospatial mapping to identify "hotspot" areas with high TB prevalence and high prevalence-to-notification ratios. Identifying these areas, facilitates efficient resource allocation and targeted case finding activities, which can impact national TB transmission rates.^8^

To translate the Initiative from policy into practice, comprehensive guidelines, aligned with the legal framework of Nepal's Public Health Service Act, 2075 were produced.^9^ The guidelines promoted a microplanning approach, where local communities develop context-specific plans to address TB based on problem analysis, cause identification and prioritization of activities and resources.^10-12^ During a high-level meeting on the TB response in the Southeast Asia Region co-hosted by Nepal, India and Indonesia, Nepal reaffirmed its commitment to ending TB locally, regionally and globally, committing to introduce Initiative activities in 25 selected municipalities.^13, 14^

Here, we present key lessons learned during implementation of the Initiative to provide insights for policymakers, program managers, and the global TB community.

To evaluate and address potential shortcomings in the implementation of the Initiative, the NTP conducted a year-long consultative process including desk-based reviews, stakeholder workshops, field-testing and disseminating guidelines.

To expedite implementation, the Nepal TB Control Center (NTCC), the apical body of the NTP, organized a national-level orientation on the Initiative with inclusive participation from the Ministry of Health and Population, technical partners (Save the Children, WHO, USAID), and provincial and municipal authorities. Following the orientation, several activities were launched. First, municipalities recruited TB Free Nepal Initiative coordinators and Monitoring and Evaluation assistants to oversee Initiative activities. Second, NTCC organized training on the implementation of the Initiative guidelines and activities at the provincial level, to which TB focal persons from Health Directorate, Health offices and partners were also invited. Third, trained staff organized an orientation for elected local TB stakeholders termed "END TB committee" members. Fourth, microplanning was conducted at the lowest administrative level and plans compiled to create a municipality-level TB plan. Fifth, priority Initiative activities including active case finding, preventive treatment and community engagement were expanded across municipalities. Finally, progress was recorded in pre-designed Initiative templates. To systematically collect data and track progress from municipalities implementing the TB-Free Initiative, NTCC developed a Google Sheet with key indicators as a regular program monitoring. This sheet was shared with designated focal points for the initiative in each municipality. These focal points were responsible for recording and updating the progress in the shared sheet, ensuring real-time data entry and transparency. Once the data was submitted, the TB-Free Initiative Secretariat Team at NTCC compiled, analyzed, and interpreted the information. The researchers analyzed and interpreted retrospectively collected data along with other routine information documented in the NTP system. The findings were then used to assess the overall progress of the initiative and were subsequently documented and presented in the manuscript, providing valuable insights for program evaluation and future planning.

## RESULTS

Significant progress has been achieved in restructuring Nepal's TB response across governmental levels. A notable development is the establishment of 112 END TB committees at municipality level and 110 municipalities formed ward level END TB committees (more than 1,000 wards) with defined roles and responsibilities to oversee the Initiative. Mobilisation of local funds, supplemented by multisector partners and municipalities, has been instrumental in supporting activities. In the 2022/23 fiscal year, municipalities and provincial health authorities allocated USD 122,000, demonstrating financial commitment. Additionally, the Initiative secured a doubling in funding for 2023/24, attributed to effective advocacy efforts.

Ensuring quality of service delivery has been a priority, 63 (56%) municipalities formed and facilitated through community led monitoring and evaluation groups to identify pathways to improvement. 16 municipalities conducted social audit on perspectives of TB-affected people has enhanced TB service delivery. TB screening by "TB Free Community Volunteers" in 53 municipalities and family-based DOTS have been introduced, enhancing TB preventive therapy accessibility, where 458 eligible children received preventive therapy through TB free initiative.

A wide range of local stakeholders have been sensitized about the Initiative, leading to increased interest, commitment, and potential impact. Review workshops in June 2023 provided a platform for multisectoral partners to assess challenges and achievements. Challenges included inadequate funding, political commitment, planning, monitoring, evaluation, logistics, and human resources for comprehensive support. Achievements included wider provision technical support, monitoring and supervision visits, and funding for community level activities to create inclusive and supportive environments for TB-affected people.

This progress and dedication underscore Nepal's proactive approach to maximize the impact of the Initiative in future years to end TB.

## CHALLENGES AND SOLUTIONS

The issues, barriers and enablers to implementation of the Initiative are summarized (Table 1).

**Table 1:** Issues, barriers and enablers for implementation of the TB Free Nepal Initiative

Issue	Barriers	Enablers
Funding and political commitment	Overall budget available from both government and other partners was insufficient to implement the TB Free Nepal Initiative Additional financial investment is required	High level of political commitment with adoption of the WHO End TB Strategy targets and endorsement of the TB Free Nepal Initiative within the Public Health Act Ringfenced domestic federal funding, including within Ministry of Health and Population budget for NTP International donors (WHO, Global Fund and USAID) willing to engage in the Initiative, with further dialogue regarding future funding ongoing
Planning, monitoring, evaluation and Logistics (including person power and staffing levels)	Difficulties recruiting and retaining staff for the initiative Transfer staff involved in implementation of the Initiative, including Chief Administrative Officer, which hampered sustainability and limited continuity of implementing team Inadequate monitoring, supervision and technical support from TB Free Secretariat at the NTCC due to shortage of human resources No active participation of elected representatives and other local level stakeholders in the TB microplanning	Local level elected representatives committed to the Iinitiative Increased multistakeholder engagement through the formation of intersectoral, interdisciplinary committees Local and municipal level resource generation (through local revenue or budget allocated at federal level for distribution at local level) to be ringfenced for the Initiative and distribution and allocation of budget to be decided at the local level Support from NTCC, province and district TB focal persons to train and build capacity of health personnel engaged in the Initiative at the municipal level through national and provincial level orientation sessions, TB management training, and direct peer supervision
TB identification and diagnosis	No provision and use of chest X-ray in TB screening camps at the community Interrupted supply of Gene Xpert cartridges and delay in replacement of modules Challenges to ensure continuity of engagement of TB Free Community Volunteers due to limited funding Difficult to identify and reach mobile underserved populations including seasonal migrants across Nepal-India border	Door to door mobilization of TB Free Community Volunteers reaching the presumptive TB cases early in the community TB Free Community Volunteers are from the local communities, which have been supportive of locating the high-risk groups and hotspot area mapping in their communities Ongoing expansion of availability of molecular WHO-approved rapid diagnostic (mWRD) centres and capacity in Nepal
Treatment, Care and Support	Budget insufficiency to provide nutritional support to all people with TB on treatment	Nutritional support provided to the poor and needy patients was perceived to reduce loss-to-follow up, promote adherence, and potentially reduce TB stigma in the community Youth group, patient support group and civil society organizations' engagement perceived as supportive to people affected by TB

## FUNDING AND POLITICAL COMMITMENT

The Initiative strengthened political commitment and mobilised funding, with 100 more municipalities expressing interest. Challenges lie in sustaining funding due to political instability and reduced governmental budgets. Successful strategies include domestic resource pooling and securing a special grant from the National Planning Commission. The initiative was presented at a (United Nations High-Level Meeting) UNHLM preparatory meeting in India, garnering international support for a sustainable financing model to end TB in the South East Asia Region. To maintain progress, exploring additional donor funds and harmonizing existing resources is essential.

## PLANNING, MONITORING, AND EVALUATION

Active engagement of elected representatives and stakeholders, delays and shortcomings in hiring and training of staff, unregulated public-private mix including access to over-the counter TB drugs, and rigorous data collection were major challenges to community level micro-planning.

However, 107 (95%) municipalities prepared ward level plans to address the TB issues. To overcome these, it was suggested to: refine the micro-planning data collection template, involve NGOs and private health care providers, and strengthen community-level organisations' micro-planning capacity; and engage the private sector, ensure timely local budget disbursement, and provide regular quality training, monitoring, and supervision to local staff. These recommendations are aimed at empowering municipal authorities in planning and evaluation and fostering an enabling environment to improve implementation of the Initiative.

## TB DIAGNOSIS

Lack of knowledge on TB and persistent stigma negatively impact access to TB services, causing delayed diagnosis and adverse health and socioeconomic outcomes. Bridging this gap requires extensive community sensitization and awareness campaigns, co-developed with TB-affected communities. Tailoring these campaigns to diverse local contexts will empower underserved populations to make autonomous health choices and will likely have synergistic positive effects on TB knowledge and stigma.

Identifying "TB hotspots", especially in plains and western hilly areas characterized by seasonal economic migration across the Nepal-India boarder, is a key challenge complicating TB screening and care efforts. Engaging TB Free Community Volunteer to reach such mobile populations migrant and improving coordination with neighbouring Indian NTP branches is crucial.

Moreover, the complex private-public healthcare system in Nepal contributes to high costs and attrition across the TB diagnostic and treatment pathway. Enhancing logistical processes, including increasing supplies of essential testing materials (GeneXpert cartridges), access to X-rays, sample transportation, strategic staff recruitment and training, and motivational incentives are vital to optimize TB diagnostic efforts and improve staff morale.

In 2023, the National Tuberculosis Program (NTP) successfully diagnosed and enrolled 40,194 individuals for TB treatment. Among these, the TB-Free Initiative played a significant role in case detection through innovative interventions. It contributed 1,790 Pulmonary Bacteriologically Confirmed (PBC) TB cases from the sputum courier program, 554 cases from screening camps and door to door visits, and 222 cases identified through primary health care/ outreach clinics (PHC/ORC). This contribution accounts for a 6% increase in the annual TB case notification, demonstrating the initiative's crucial impact in strengthening TB detection and control efforts. Such a contribution significantly enhances the NTP's overall efforts to improve case detection, early treatment initiation, and ultimately reduce the TB burden in Nepal.

## TB CARE AND SUPPORT

Holistic counselling, reliable medicine supply, and vigilant monitoring are cornerstones of TB care. The initiative has improved support to people with TB from within their household or family, including designating family members as treatment supporters for family-based DOTS, 23 forming patient support groups for TB-affected people, and engaging 32 youth groups and 32 civil-society organisations (CSOs). These efforts aim to increase morale, reduce stigma, and improve TB diagnosis, treatment, and prevention outcomes. However, nutritional support, including cash transfers and food baskets for under-served individuals with TB with low BMI/food insecurity, the budget has not yet been secured to make nutritional support universal. Introducing a comprehensive TB-affected family counselling package, covering stigma, costs, and nutrition, could further strengthen existing Initiative measures.

## LESSONS LEARNT AND CONCLUSION

Since its implementation in 2021, the TB Free Nepal Initiative has made major advances in identifying missing TB cases and has the potential to contribute to ending TB in Nepal. The Initiative has appropriate ownership by local level governments committed to ending TB in Nepal. Capacity enhancement, microplanning, and supportive monitoring have been crucial to successfully implement the Initiative. Proper guidance from the federal level and partnership with the provincial level to provide technical assistance support to the local level requires strengthening. Door-to-door mobilization of the TB Free Community Volunteers for presumptive TB identification has proven as effective interventions. Engagement of youth groups, CSOs, TB-affected individual's groups, and local volunteers has diversified the TB response efforts. The initiative scale-up is ongoing with supported by detailed monitoring, evaluation and multisectoral dialogue about results, lessons learned. The TB Free Nepal Initiative has been successful in developing locally-appropriate strategies and guidelines and remains committed to Nepal's goal of ending TB by 2050. Based on the observations, the following recommendations have been made to strengthen the initiative in the future.

- The TB Free Nepal Initiative is instrumental to finding missing TB cases in the community
- Effective community engagement is vital to successfully implement the Initiative
- A shift from supply side to demand side program planning, implementation, participatory monitoring and evaluation can enable increased political commitment and effective participation of private sectors
- TB micro planning is a foundation of the Initiative and facilitates to increase ownership, partnership and accountability of local representative and communities.
